# Enhanced 1.54-μm photo- and electroluminescence based on a perfluorinated Er(III) complex utilizing an iridium(III) complex as a sensitizer

**DOI:** 10.1038/s41377-020-0266-3

**Published:** 2020-03-04

**Authors:** Hong-Fei Li, Xiao-Qi Liu, Chen Lyu, Jelena Gorbaciova, Li-Li Wen, Guo-Gang Shan, Peter. B. Wyatt, Huan-Qing Ye, William P. Gillin

**Affiliations:** 1 Materials Research Institute and School of Physics and Astronomy, Queen Mary University of London, Mile End Road, E1 4NS London, UK; 2 Institute of Functional Material Chemistry, Faculty of Chemistry, Northeast Normal University, Changchun, 130024 China; 3 Materials Research Institute and School of Biological and Chemical Sciences, Queen Mary University of London, Mile End Road, E1 4NS London, UK; 4 Chromosol Ltd, The Walbrook Building, 25 Walbrook, London, EC4N 8A UK

**Keywords:** Optics and photonics, Organic LEDs

## Abstract

Advanced 1.5-µm emitting materials that can be used to fabricate electrically driven light-emitting devices have the potential for developing cost-effective light sources for integrated silicon photonics. Sensitized erbium (Er^3+^) in organic materials can give bright 1.5-µm luminescence and provide a route for realizing 1.5-µm organic light emitting diodes (OLEDs). However, the Er^3+^ electroluminescence (EL) intensity needs to be further improved for device applications. Herein, an efficient 1.5-µm OLED made from a sensitized organic Er^3+^ co-doped system is realized, where a “traditional” organic phosphorescent molecule with minimal triplet–triplet annihilation is used as a chromophore sensitizer. The chromophore provides efficient sensitization to a co-doped organic Er^3+^ complex with a perfluorinated-ligand shell. The large volume can protect the Er^3+^ 1.5-µm luminescence from vibrational quenching. The average lifetime of the sensitized Er^3+^ 1.5-µm luminescence reaches ~0.86 ms, with a lifetime component of 2.65 ms, which is by far the longest Er^3+^ lifetime in a hydrogen-abundant organic environment and can even compete with that obtained in the fully fluorinated organic Er^3+^ system. The optimal sensitization enhances the Er^3+^ luminescence by a factor of 1600 even with a high concentration of the phosphorescent molecule, and bright 1.5-µm OLEDs are obtained.

## Light emission: Erbium and iridium make a powerful combination

Light emission from ions of the element erbium, for use in electrically driven light-emitting devices, can be significantly enhanced by adding sensitizer materials containing iridium ions. The potential of erbium for cost-effective light emission in photonics applications is well-recognized, but methods to increase the power efficiency of the devices are keenly sought. Hong-Fei Li and colleagues at Queen Mary University of London, UK, and Northeast Normal University in Changchun, China, found that a phosphorescent iridium complex enhanced the emission from an erbium complex by a factor of up to 1600. The dramatic effect of the iridium sensitizer was demonstrated in an organic (carbon-based) light-emitting diode. This innovation emphasizes the potential advantages of exploring combinations of molecular complexes in light-emitting devices, rather than seeking a single light-emitting molecule possessing all of the properties required.

## Introduction

The Er^3+^ ion has been of importance in photonic applications because its ~1.5-µm emission matches the low-loss C-band telecommunication window^1–7^. Direct photoexcitation of Er^3+^ ions to produce population inversion requires high power densities due to the weak optical absorption of the partially forbidden 4 f electron transitions. Enhancing the power efficiency of excitation for Er^3+^ ions is highly desired for advanced applications, especially for cost-effective light sources in silicon photonic systems used for telecommunications^7^. Incorporating Er^3+^ ions into an environment with organic light-harvesting chromophores can significantly enhance the erbium emission through sensitizations of organic excitons onto Er^3+^ excitations^7–12^. The intense absorption of organic chromophores and subsequent sensitizations allow for population inversion of Er^3+^ ions at a power density of <1 W/cm^2^, compared with ~100 kW/cm^2^ required by any inorganic Er^3+^-doped material. In addition, organic semiconductor material hosts allow for electrically driven 1.5-µm EL devices and even 1.5-µm lasers and amplifiers that can be integrated onto silicon photonic circuits^7–9,13–15^. The sensitization can be attributed to both singlet and triplet excitons of organic chromophores, while triplet excitons^8^ are frequently cited to provide more efficient sensitization for Er^3+^ excitations than singlet excitons^9^. Triplet excitons can persist with long phosphorescence lifetimes, and these low transition rates provide less competition for the energy transfer (ET) to the Er^3+^ ions. Furthermore, their long diffusion lengths favour ET to those Er^3+^ ions that are distant from chromophores. Compared with organic compounds and fluorescent metal complexes, the UV-vis absorption of phosphorescent metal complexes for Er^3+^ sensitization originates not only from metal-to-ligand-charge-transfer (MLCT) but also from ligand-to-metal-charge-transfer (LMCT), ligand-to-ligand-charge-transfer (LLCT) and π–π* transitions (LC). Their energy levels are low enough to match those for the Er^3+^ ion in the visible region. By utilizing a phosphorescent complex with a long triplet lifetime as a sensitizer to replace a fluorescent complex, the sensitizing factor of Er^3+^ ions can be further increased. Since the heavy-metal-atom effect increases spin-orbital coupling and enhances intersystem crossing to generate triplet excitons, some organometallic phosphorescent materials have been considered sensitizers^8^. For example, phosphorescent Ir(III) complexes have attracted much attention as both singlet and triplet excitons are emissive, and they can reach an internal quantum efficiency of 100% and an external quantum efficiency over 20%^16,17^, making them promising candidates for Er^3+^ complex sensitizers in near-infrared (NIR) EL applications. Li et al. reported NIR photoluminescence (PL) and EL based on tris-(dibenzoylmethanato)-mono-(bathophenanthroline) erbium (Er(DBM)_3_bath) sensitized by tris[2-phenylpyridinato-C^2^,N]iridium(III) (Ir(ppy)_3_); however, it only enhanced Er^3+^ PL and EL by a factor of 20 and 4, respectively, and required a high drive voltage to produce weak 1.5-µm EL^8^. Part of the reason for such poor performance is that triplet–triplet annihilation (TTA) within the chromophore rapidly quenches the triplet excitons and consequently reduces the population available for sensitization. In addition, those organic complexes contain many C–H bonds, which would cause severe vibrational quenching, leading to a low efficiency for the 1.5-µm luminescence^18–21^.

In this work, we successfully demonstrate considerable sensitization for the Er^3+^ ion in a fully fluorinated complex Er(F-TPIP)_3_ by taking an organic phosphorescent complex Ir-tBuPBI as a sensitizer. Er(F-TPIP)_3_ is an Er(III) complex of tetrakis(pentafluorophenyl)imidodiphosphinate, and Ir-tBuPBI is a six-coordinate Ir(III) species bis[5-*tert*-butyl-2-(1-phenyl-1*H*-benzo[*d*]imidazol-2-yl-κN^3^)phenyl-κC](5-(pyridin-2-yl-κN)-3-trifluoromethyl-1*H*-1,2,4-triazol-1-yl-κN)iridium. Ir-tBuPBI and Er(F-TPIP)_3_ are composited at the molecular level using co-evaporation. Ir-tBuPBI is chosen as the sensitizer because it exhibits a low TTA rate and efficiency roll-off but higher efficiency of PL and EL in a neat film than other non-doped Ir(III) materials, such as Ir(ppy)_3_^22^. These features allow Ir-tBuPBI to provide efficient sensitization even at high concentrations. Er(F-TPIP)_3_ molecules have perfluorinated ligands and a large volume, ~2004 Å^3^, determined from the single crystal structure. This enclosure keeps the C–H bonds on Ir-tBuPBI distant from the central Er^3+^ ion to reduce vibrational quenching^23–26^. Photoexcitation gives a sensitization factor of 1600 for the Er^3+^ 1.5-µm PL, two orders of magnitude larger than the best reported results for a non-halogenated phosphorescent chromophore^8^, and the longest Er^3+^ NIR emission lifetime in an organic hydrogenated environment is obtained. Furthermore, intense 1.5-µm EL is observed from an OLED fabricated using such a co-doped Ir-tBuPBI:Er(F-TPIP)_3_ film as the emissive layer.

## Results

### Photophysical properties

**Fig. 1:**
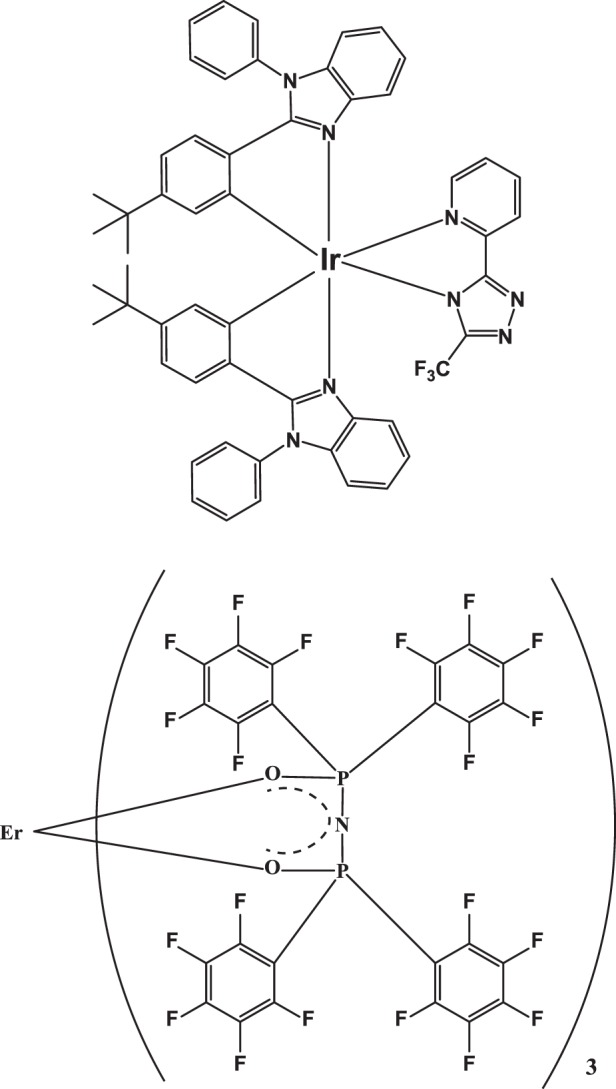
Chemical structures of the materials. Chemical structures of Ir-tBuPBI (top) and Er(F-TPIP)_3_ (bottom)

**Fig. 2:**
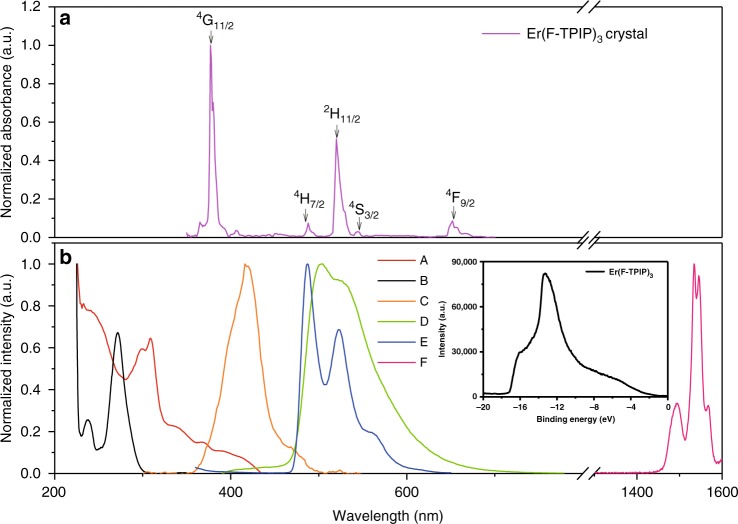
Excitation, absorption and photoluminescence spectra. **a** The absorption spectrum for a single crystal of Er(F-TPIP)_3_. **b** The absorption spectra of Er(F-TPIP)_3_ in DMSO (A) and Ir-tBuPBI in CH_2_Cl_2_ at room temperature (B). The excitation spectrum of an Er(F-TPIP)_3_: Ir-tBuPBI co-doped film recorded at 1532 nm (C). The emission spectra of the Ir-tBuPBI film at room temperature (D) and Ir-tBuPBI in CH_2_Cl_2_ at 77 K (E). The NIR emission spectrum of a co-doped film of 50% Er(F-TPIP)_3_: 50% Ir-tBuPBI (F). The UPS spectrum of Er(F-TPIP)_3_ (Inset)

The chemical structures of Er(F-TPIP) and Ir-tBuPBI are shown in Fig. 1. Their photophysical properties are studied, and the spectra are plotted in Fig. 2a, b. The absorption spectrum of Er(F-TPIP)_3_ dissolved in DMSO exhibits two strong absorption bands with peaks at 236 and 271 nm. These bands are consistent with the absorption of F-TPIP^−^, and they are thus attributed to the ligand π–π* transition^25^. The energy gap of Er(F-TPIP)_3_ can be determined as 4.1 eV through its absorption edge localized at 301 nm. The ultraviolet photoelectron spectroscopy (UPS) spectrum shown in the inset of Fig. 2 predicts that the HOMO level of Er(F-TPIP)_3_ is –5.9 eV and the LUMO level is –1.8 eV. Ir-tBuPBI in dichloromethane solution at room temperature shows a strong absorption band at 310 nm, which is mainly attributed to the spin-allowed ligand-centred π–π* transition. The weak absorption band at wavelengths longer than 340 nm in the lower energy region can be assigned to the LC, MLCT and LLCT transitions of Ir-tBuPBI^22^. The absorption spectrum of an ~100-µm-thick Er(F-TPIP)_3_ crystal is shown in Fig. 2a, and it can be seen that the three absorption bands of ^4^F_7/2_, ^2^H_11/2_ and ^4^S_3/2_ of the Er^3+^ ion in Er(F-TPIP)_3_ perfectly match the emission bands of Ir-tBuPBI in CH_2_Cl_2_ at 77 K. The good spectral overlap between the Ir-tBuPBI emission bands and the absorption bands of the Er^3+^ ions means that efficient sensitization may be realized through ET from the ^3^LC, ^3^MLCT and ^3^LLCT levels of Ir-tBuPBI to the ^4^F_7/2_, ^2^H_11/2_ and ^4^S_3/2_ energy levels of the Er^3+^ ions. An excitation spectrum of an Er(F-TPIP)_3_:Ir-tBuPBI (1:1) co-doped film recorded at 1532-nm emission overlaps the lower energy region of the Ir-tBuPBI absorption spectrum very well, and no overlap between this excitation spectrum and the absorption spectrum of Er(F-TPIP)_3_ solution is found; thus, the 1.5-µm emission from Er^3+^ ions is solely due to the ET from Ir-tBuPBI to Er(F-TPIP)_3_.

### Sensitization effect

**Fig. 3:**
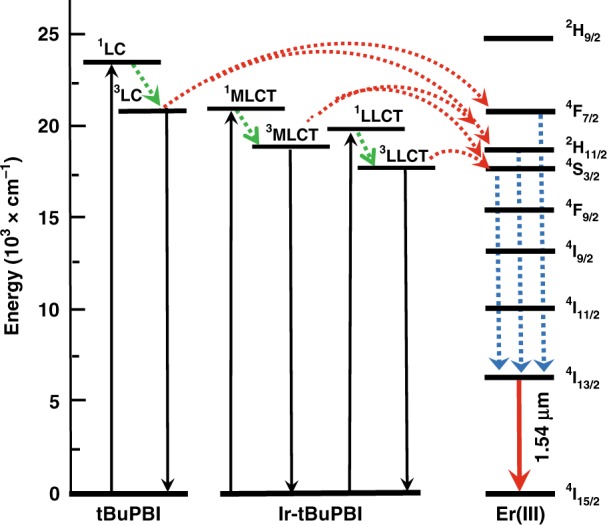
The Jablonski diagram of intermolecular energy transfer from Ir-tBuPBI to Er(F-TPIP)_3_ for NIR emission. Red dashed lines represent a Förster procedure

Generally, there are two possible types of ET mechanisms between chromophore molecules and Er^3+^ ions: Dexter and Förster energy transfer. Dexter transfer preserves the total spin of the system and thus allows triplet–triplet ET from donor to acceptor. It is a short-range process requiring wave-function overlap between the donor and acceptor^27^. Therefore, direct Dexter energy transfer from the donor Ir-tBuPBI to the acceptor Er^3+^ ion is unlikely because the Er^3+^ ion is well isolated by the F-TPIP ligands, which prevents coordination of Er^3+^ ions with Ir-tBuPBI. The absorption of the F-TPIP ligand has no overlap with the phosphorescence spectrum of donor Ir-tBuPBI. Therefore, Dexter energy transfer from Ir-tBuPBI to the F-TPIP ligands in Er(F-TPIP)_3_ as an intermediary and then on to the Er^3+^ ions can also be ruled out. However, there is a perfect overlap between the phosphorescence emission of Ir-tBuPBI and the absorption peaks of the Er^3+^ ion in the Er(F-TPIP)_3_ single crystal. This means that Förster energy transfer from the triplet states of the Ir-tBuPBI to Er^3+^ ions may occur. Förster energy transfer is a long-range process with nanometre-scale distances. Hence, we estimate the Förster radius (*R*_0_), which is defined as the distance between a sensitized Er^3+^ ion centre and an excited Ir-tBuPBI centre at which the ET efficiency is 50%. *R*_0_ depends on the overlap integral of the donor emission spectrum with the acceptor absorption spectrum and their mutual molecular orientation. We calculate the *R*_0_ for the triplet of Ir-tBuPBI to couple to the Er^3+^ ion by the following equation.

$$R_0^6 = \frac{{9000\ln 10}}{{128\pi ^5N_{\mathrm{A}}}}\frac{{\kappa ^2Q_{\mathrm{D}}}}{{n^4}}{\int} {f_{\mathrm{D}}(\lambda )\varepsilon _{\mathrm{A}}(\lambda )\lambda ^4d\lambda }$$

where *N*_A_ is Avogadro’s number, *k*^2^ is the dipole orientation, *Q*_D_ is the quantum yield of the donor, *n* is the refractive index of the medium, *f*_D_(*λ*) is the normalized donor emission spectra and *ε*_A_(*λ*) is the acceptor molar absorption coefficient. Given these terms, a Förster radius of ~18 Å is obtained. This suggests that Förster energy transfer from the triplet states of Ir-tBuPBI to the corresponding energy levels of Er^3+^ ions is the likely main ET mechanism. The Jablonski diagram of the ET routes is depicted in Fig. 3.

**Fig. 4:**
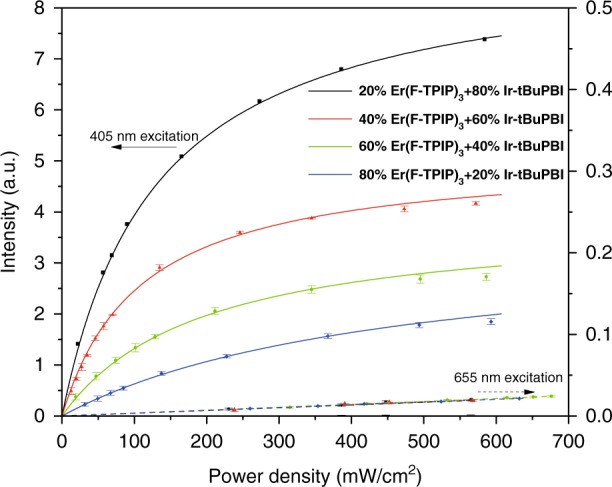
The emission intensity per percentage Er^3+^ at 1532 nm as a function of laser power density for Ir-tBuPBI co-doped film with different molecular concentrations of Er(F-TPIP)_3_. The solid line represents 405-nm excitation, and the dashed line represents 655-nm excitation

To quantify the sensitization factor, 405- and 655-nm diode lasers are used to excite the Er^3+^ ions either via the chromophore or directly, and the power-dependent Er^3+^ PL intensities at the two excitation wavelengths are compared. The power-dependent PL intensities of these co-doped films that contain Er(F-TPIP)_3_ molecules with molecular percentages of 20, 40, 60 and 80% recorded at an emission wavelength of 1532 nm are shown in Fig. 4, where the absolute PL intensities are normalized to the Er^3+^ concentration percentages in those films. The 655-nm photoexcitation directly excites the Er^3+^ ions to the ^4^F_9/2_ level, and the power-dependent PL spectra are identical because the intrinsic absorption cross-section of the ^4^I_15/2_ → ^4^F_9/2_ transition is independent of the Er(F-TPIP)_3_ concentration. The weak absorption requires an excitation power density of over 250 mW/cm^2^ to obtain detectable Er^3+^ PL from the films. However, 405-nm photoexcitation greatly enhances Er^3+^ PL by exciting Ir-tBuPBI to sensitize Er^3+^ excitation. The overlap of the ^4^I_15/2_ → ^2^H_11/2_ absorption with the Ir-tBuPBI emission allows Er^3+^ ions to be populated, particularly through the hypersensitive ^4^I_15/2_ → ^2^H_11/2_ transition that provides a strong oscillator strength to the dipole–dipole interaction with the Ir-tBuPBI excitons.

The sensitisation factor is defined as an effective enhancement factor in the emission intensity for light absorbed by the chromophore compared with the direct absorption into the ^4^I_15/2_ → ^4^F_9/2_ transition. The sensitization factors per Er^3+^ percentage are found to be dependent on the Er(F-TPIP)_3_ doping concentration, and the values are obtained through modelling rate equations with the power-dependent Er^3+^ PL spectrum at 405-nm photoexcitation by taking 655-nm photoexcitation as a ref. ^28^. This gives sensitization factors of 1600, 1200, 600 and 210 for the 20%, 40%, 60% and 80% doped Er(F-TPIP)_3_ films, respectively.

### Time-resolved PL studies for energy transfer

**Fig. 5:**
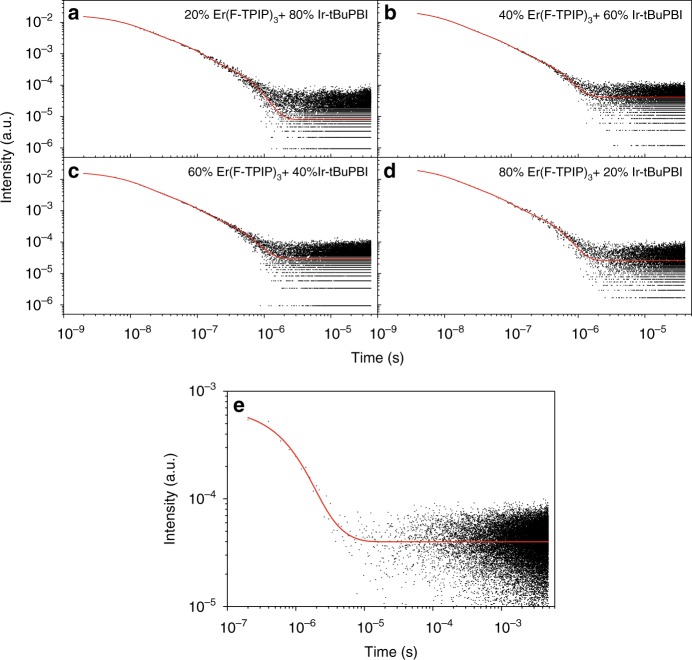
Time-resolved PL spectra for 520 nm emission. The TRPL decay (*λ*_mon_ = 520 nm) of the co-doped samples with Er(F-TPIP)_3_ concentration ranging from 20 to 80% (**a**–**d**) and a neat Ir-tBuPBI film (**e**) excited under a 430-nm OPO laser

The time-resolved PL (TRPL) decay curves for the Ir-tBuPBI emission recorded at 520 nm (*λ*_mon_ = 520 nm) with different concentration ratios of Er(F-TPIP)_3_ and Ir-tBuPBI are shown in Fig. 5a–e, and the corresponding emission lifetimes are listed in Table 1. The Ir-tBuPBI emission lifetime, $\tau {^\prime}$, remains similar when the concentration of Er(F-TPIP)_3_ increases from 20 to 80%. Specifically, the results show that $\tau {^\prime}$ is ~0.155 ± 0.001 μs for Er(F-TPIP)_3_ concentrations from 20 to 60% and decreases slightly to ~0.144 ± 0.001 μs at 80% Er(F-TPIP)_3_. In the neat Ir-tBuPBI film, this lifetime reaches ~1.2 ± 0.13 µs, which is longer than that in any of the co-doped films. It is known that Ir-tBuPBI reduces TTA-based self-quenching. Thus, the shorter lifetimes demonstrate ET from Ir-tBuPBI excitons to Er^3+^ excitations, which reduces the Ir-tBuPBI emission lifetime. Thus, an overall ET rate (*R*_ET_) can be calculated by the formula of $R_{ET} = 1/\tau {^ \prime} - 1/\tau$, where $\tau {^\prime}$ indicates the Ir-tBuPBI emission lifetime in the neat film and *τ* indicates the one in the presence of Er(F-TPIP)_3_. The calculation gives overall ET rates of 5.7 × 10^6^ s^−1^ and 6.1 × 10^6^ s^−1^ for the 20 and 80% Er (F-TPIP)_3_ concentrations, respectively. It is noteworthy that the average distance between an Ir-tBuPBI sensitizer and its closest-surrounding Er(F-TPIP)_3_ molecules should be similar due to the uniformity of codoping of the two molecules in this system. Hence, for an energy transfer process, the closest molecules should dominate ET. On the other hand, it is clear that the increase in the Er(F-TPIP)_3_ concentration decreases the Ir-tBuPBI concentration. Considering that the TTA-related self-quenching of Ir-tBuPBI is known to be minimal, its emission lifetime would not be greatly affected by its concentration. Therefore, these facts explain why the Ir-tBuPBI emission lifetime and the ET rate are similar in those samples with increasing Er(F-TPIP)_3_ concentration and decreasing Ir-tBuPBI concentration, respectively.

**Table 1:** The average lifetimes of Ir-tBuPBI emission for the co-doped films and neat Ir-tBuPBI film excited under a 430-nm OPO laser

Samples	$\tau {^\prime}$ (µs)
20% Er(F-TPIP)_3_:80% Ir-tBuPBI	0.155 ± 0.001
40% Er(F-TPIP)_3_:60% Ir-tBuPBI	0.155 ± 0.001
60% Er(F-TPIP)_3_:40% Ir-tBuPBI	0.153 ± 0.001
80% Er(F-TPIP)_3_:20% Ir-tBuPBI	0.144 ± 0.001
100% Ir-tBuPBI	1.2 ± 0.1

**Fig. 6:**
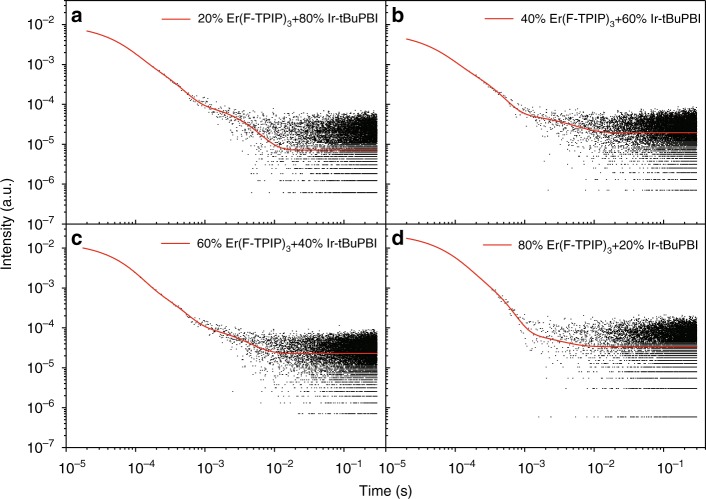
Time-resolved PL spectra at 1535 nm emission. The TRPL decay (*λ*_mon_ =1532 nm) of the co-doped samples with Er(F-TPIP)_3_ concentrations ranging from 20 to 80% (**a**–**d**) when excited under a 430-nm OPO laser

The results of TRPL measurements for the Er^3+^ emission lifetime with different Er(F-TPIP)_3_ and Ir-tBuPBI concentrations are shown in Fig. 6a–d. The values of their longest component (*τ*_L_), average lifetime (*τ*_Ave_) and the photoluminescence quantum yields (PLQYs) of Er^3+^ are listed in Table 2. The 20% Er(F-TPIP)_3_ co-doped film gives an average lifetime of 867 ± 14 μs for the Er^3+^ 1.5-µm emission, which is by far the longest recorded for a system that contains hydrogenated bonds and is even longer than that of a fully halogenated Er^3+^-doped system^7^. There are three lifetime components of ~34.0 ± 0.1 µs, 200 ± 14 µs and 2.65 ± 0.1 ms. The shortest component is the system response time, and the middle component is comparable with the reported emission lifetime for the Er(F-TPIP)_3_ film measured in air^23^. However, the ~2.65 ms lifetime, which accounts for almost 30% of the emission, is unexpectedly long in the environment of co-doped Ir-tBuPBI molecules that contain a number of C–H bonds. It is noteworthy that this ~2.65 ms lifetime is 12 times longer than that of neat Er(F-TPIP)_3_ (0.2 ms)^23^ and is longer than that of non-halogenated Er(TPIP)_3_ (2.3 µs) by a factor of 1100^29^. Although the lifetime measurements of the Ir-tBuPBI emission give an emission lifetime of 0.15 µs, some persistent triplet states may still be present (but below the detector sensitivity), particularly since the Ir-tBuPBI emission has never been confirmed to have unity efficiency. Thus, it is likely that this long lifetime is attributable to the coupling of residual persistent triplet excitons to Er^3+^ ions with a low ET rate, leading to persistent Er^3+^ emission. In fact, the phosphorescence lifetime of organic Ir(III) complexes is reported to vary in a wide range from a hundred nanoseconds to several microseconds^30,31^, with some Ir(III) complexes demonstrating luminescence lifetimes as long as 4 ms^32^.

**Table 2:** The Er^3+^ 1.5-µm emission average lifetimes, the longest lifetime component and PLQY of the Er^3+^ ions in the co-doped films excited under a 430-nm OPO laser

Samples	Longest component *τ*_L_ (ms)	Average *τ*_Ave_ (µs)	PLQY (%)
20% Er(F-TPIP)_3_:80% Ir-tBuPBI	2.65 ± 0.1 (30.82%)	867 ± 14	10.84
40% Er(F-TPIP)_3_:60% Ir-tBuPBI	2.63 ± 0.15 (24.14%)	723 ± 17	9.04
60% Er(F-TPIP)_3_:40% Ir-tBuPBI	2.47 ± 0.09 (23.04%)	650 ± 16	8.13
80% Er(F-TPIP)_3_:20% Ir-tBuPBI	2.24 ± 0.08 (5.65%)	246 ± 20	3.08

With the increase in Er^3+^ concentration, the longest Er^3+^ emission lifetime decreases slightly from 2.65 ± 0.1 ms to 2.24 ± 0.08 ms; with the lifetime component percentages declining dramatically from ~30 to ~5%, the average lifetime (*τ*_Ave_) decreases from 867 ± 14 to 246 ± 20 µs; and the PLQY values of Er^3+^ ions are reduced from 10.84 to 3.08%. This indicates that the increased Er^3+^ concentration is more effective in quenching the triplet states and hence removing the long-lived ET route.

### EL properties

**Fig. 7:**
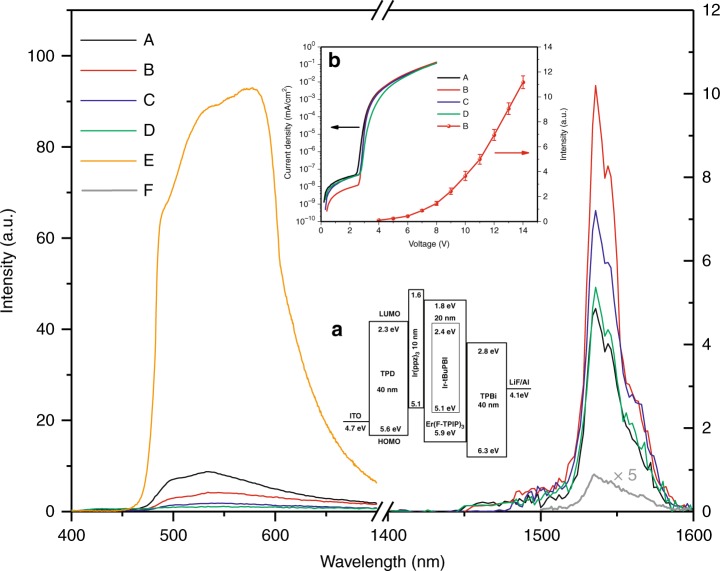
The EL spectra of OLEDs with different molecular ratios of Ir-tBuPBI:Er(F-TPIP)3 driven with 14 V. 5% Er(F-TPIP)_3_ + 95% Ir-tBuPBI (A), 10% Er(F-TPIP)_3_ + 90% Ir-tBuPBI (B), 30% Er(F-TPIP)_3_ + 70% Ir-tBuPBI (C), 50% Er(F-TPIP)_3_ + 50% Ir-tBuPBI (D), 100% Ir-tBuPBI (E) and 10% Er(F-TPIP)_3_ + 90% AlQ_3_ (F). Inset a: Schematic energy level diagram of the OLED used in this study. Inset b: The current density-voltage characteristics for the co-doped OLEDs with different molecular ratios of Ir-tBuPBI:Er(F-TPIP)_3_ and the EL intensity-voltage characteristics for the 10% Er(F-TPIP)_3_:90% Ir-tBuPBI device

NIR EL devices using a neat Er(F-TPIP)_3_ film as the emissive layer are studied, and no NIR emission is observed. The sensitization and the phosphorescent properties of Ir-tBuPBI allow for the fabrication of Er-based 1.5-µm emitting OLEDs with a structure of ITO/*N*,*N*′-bis(3-methylphenyl)-*N*,*N*′-diphenylbenzidine (TPD) (40 nm)/tris(phenylpyrazole)iridium [Ir(ppz)_3_] (10 nm)/Ir-tBuPBI:xEr(F-TPIP)_3_(x: molar ratio, 20 nm)/2,2′,2″-(1,3,5-benzitriyl)-tris(1-phenyl-1-H-benzimidazole) (TPBi) (40 nm)/lithium fluoride (LiF) (1 nm)/Al (100 nm), and the device structure diagram is shown in the inset (a) of Fig. 7, where TPD, Ir(ppz)_3_ and TPBi are used as the hole transporting, electron blocking, electron transporting and hole blocking layers, respectively. The inset (b) of Fig. 7 shows the current density-voltage characteristics for the co-doped OLEDs with different molecular ratios of Er(F-TPIP)_3_ and Ir-tBuPBI concentrations and the EL intensity-voltage characteristics for the 10% Er(F-TPIP)_3_:90% Ir-tBuPBI device.

The leakage currents for all devices are small, especially for the 10% Er(F-TPIP)_3_ doped device with the strongest NIR EL, which shows the lowest leakage current with an order of magnitude smaller than the other co-doped devices. The threshold voltage is found to be similar within a range between 2.45 V and 2.69 V among devices. It is found from inset b of Fig. 7 that the NIR EL from the 10% Er(F-TPIP)_3_ + 90% Ir-tBuPBI device can be detected when the drive voltage is as low as 4 V. The NIR detection is limited by the sensitivity of the detector, and hence, emission is probably occurring from even closer to the turn-on voltage of the device. The visible and NIR EL spectra of the devices with different Er(F-TPIP)_3_ molecular concentration percentages at 14 V are shown in Fig. 7. The Ir-tBuPBI in all the co-doped devices shows considerably weaker EL than that in a device with a neat Ir-tBuPBI emissive layer in the visible region. The visible emission intensity decreases as the Er(F-TPIP)_3_ concentration increases, which implies that an efficient ET from Ir-tBuPBI to Er(F-TPIP)_3_ occurs.

The Er^3+^ EL intensity can be seen to increase from an Er(F-TPIP)_3_ concentration of 5% to reach a maximum intensity at 10% Er(F-TPIP)_3_ before decreasing at higher Er^3+^ concentrations. Hereafter, 10% is used as the optimal doping level. To indicate the brightness of our device, we create an OLED for comparison by replacing 90% Ir-tBuPBI with 90% tris-(8-hydroxyquinoline) aluminium (AlQ_3_), which is a known sensitizer for Er^3+^-based OLEDs^33^. The corresponding EL spectrum is also shown in Fig. 7, and the intensity is 60 times weaker than that of the 1.5-μm EL of our OLED made from 10% Er(F-TPIP)_3_:90% Ir-tBuPBI.

Given the evidence that the sensitization factor increases with the decrease in the Er^3+^ ion concentration, the EL increase from 5 to 10% can be attributed to the increasing absolute number of Er(F-TPIP)_3_ molecules in the emissive layers rather than any increase in sensitization. When the Er(F-TPIP)_3_ concentration exceeds 10%, the Er^3+^ EL intensity starts to decrease due to decreasing sensitization. Nevertheless, for a doping concentration of 30% Er(F-TPIP)_3_, the NIR EL intensity still reaches 70% of that at the optimal doping level.

## Discussion

We demonstrate that a phosphorescent organic Ir(III) complex can be used as an optical sensitizer to enhance the Er^3+^ 1.5-µm emission from a composite co-doped with an organic Er^3+^ complex and Ir(III) complex at the molecular level. This enhancement can reach a factor of 1600 for a thin film with a 20% molecular concentration of the organic Er^3+^ complex due to the sensitization from the organic triplet excitons. The perfluorinated-ligand shell of the organic Er^3+^ complex effectively reduces the vibrational quenching, giving an average lifetime of 867 ± 14 μs for the Er^3+^ 1.5-µm emission in an environment containing abundant hydrogenated Ir-tBuPBI chromophores, which is comparable to the value of an organic fully fluorinated co-doped Er^3+^ system. Moreover, the material system facilitates the successful fabrication of a 1.5-µm emitting OLED. The device emits intense 1.5-µm EL with only a 10% Er(F-TPIP)_3_ molecular percentage in the emissive layer. This achievement is attributed to the high EL efficiency and intensity of Ir-tBuPBI, the protection of the central Er^3+^ ion by the F-TPIP ligands, and the approach of separating the function of the high-efficiency Er^3+^ emitter from that of a sensitizer molecule that allows for considerable OLED performance to make a composite device, which shows that the use of protiated phosphorescent molecules is not greatly detrimental to the quantum efficiency of the Er^3+^ ions. The demonstration highlights that future researchers can seek to optimize the individual performance of each component, rather than the current, difficult approach of seeking to design a single molecule with all the contradictory properties required.

## Materials and methods

### Materials

Ir-tBuPBI^22^ and Er(F-TPIP)_3_^23^ were prepared according to previously reported literature methods and purified by using a train vacuum sublimation technique. The single crystal of Er(F-TPIP)_3_ was prepared with the following procedure: 52.4 mg Er(F-TPIP)_3_ powder was dissolved in 3 mL dimethyl sulfoxide (DMSO). Pink crystals were precipitated for a week at room temperature, filtered off and washed with diethyl ether. All the charge-transporting and blocking materials used in the process of OLED fabrication, including TPD, Ir(ppz)_3_, TPBi and LiF, were commercially available and were used without further purification unless otherwise noted.

### Fabrication of samples for PL measurements

Co-doped films of Ir-tBuPBI (AlQ_3_) and Er(F-TPIP)_3_ were deposited by vacuum sublimation at a vacuum pressure of ~10^−7^ mbar. Then, 120-nm-thick aluminium was evaporated onto the organic layers to protect the materials from atmospheric degradation. Samples were prepared with Er(F-TPIP)_3_ molecular concentrations of 20% (50 nm), 40% (130 nm), 60% (300 nm) and 80% (770 nm). Each film had an identical amount of Ir-tBuPBI chromophore (100-nm thick) to ensure that the absorption of the 405-nm laser was constant in each film. This thickness was chosen to ensure that the light was absorbed relatively uniformly through all the films.

### Fabrication of OLEDs for EL measurements

Prepatterned ITO substrates with a resistivity of <48 Ω/sq were cleaned by sequential sonication in detergent solution, ultra-pure water, acetone and chloroform and finally blown dry with nitrogen. The cleaned ITO substrates were then treated with oxygen plasma for 5 min before being loaded into the vacuum chamber of the deposition system. The OLEDs are fabricated by successively sublimating organic and inorganic layers onto precleaned ITO-coated glass substrates in a vacuum deposition system. Organic and inorganic layers are deposited at a base pressure of 2 × 10^−8^ mbar. The thermal deposition rates for organic materials, LiF and Al are ~2, ~0.5 and 6 Å/s, respectively. The effective individual device area is 4 mm^2^. The OLED made from a co-doped emissive layer of 90% AlQ_3_ and 10% Er(F-TPIP)_3_ has a structure of ITO/TPD (40 nm)/90% AlQ_3_ + 10% Er(F-TPIP)_3_ (20 nm)/TPBi (40 nm)/LiF (1 nm)/Al (100 nm). This device excludes the Ir(ppz)_3_ layer since it is known that the HOMO and LUMO of TPD match those of AlO_3_ for effective hole injection.

## Characterization

### Absorption spectral measurements

UV-vis (ultraviolet-visible) absorption spectra were obtained using a Shimadzu UV-2600 spectrophotometer.

### Excitation spectral measurements

An Apex Arc Xenon lamp coupled to a spectrometer was used as the excitation source, and the measured excitation spectrum was calibrated by measuring the power of each wavelength using a Newport MODEL 818-UV silicon detector connected to a Newport Multi-Function Optical Metre Model 1835-C.

### Emission spectra and TRPL measurements

For the PL measurements, lasers of different wavelengths were used to excite the samples, the emission from the samples was guided into a spectrometer (Jobin Yvon Horiba Triax 550), and the reflected laser light was removed by placing high-pass filters in front of the spectrometer. For the EL measurements, the OLED was placed into a homemade OLED holder and driven with the voltage from a waveform function generator to produce an emission, and the EL emission was directed into the spectrometer. The spectrometer was connected to a photomultiplier (PMT), and the signal from PMT was transmitted to an oscilloscope or a lock-in-amplifier for time-resolved or emission spectral measurements, respectively. A Q-switch Nd:YAG laser was used to produce a pulsed laser, and the laser wavelength was tuned by using an optical parametric oscillator (OPO). Pulsed laser beams were directed incident onto co-doped films to give TRPL data. The data were fitted by using exponential functions: *I*(*t*) = *I*_0_ + ∑_*i*_ *A*_*i*_ · Exp[−*t*/*τ*_*i*_]. A lifetime component percentage is obtained by an expression of *A*_*i*_ · *τ*_*i*_*/*(∑_*i*_ *A*_*i*_ · *τ*_*i*_). An average lifetime is obtained by an expression of <*τ*> = ∑_*i*_ [*τ*_*i*_ · *A*_*i*_ · *τ*_*i*_*/*(∑_*i*_ *A*_*i*_ · *τ*_*i*_)].

### Sensitization measurements

Two apertures were used to ensure the consistency of the alignment of the optical path. A mirror made by growing an aluminium circle with a 2-mm diameter on the 20 mm × 20 mm glass substrate was set to an angle of 45° to reflect light normally onto the sample and allow the PL to be collected. An aperture with a 1-mm diameter hole was placed directly in front of the sample, and the laser was defocused to ensure uniform illumination over the whole sample. Once the optical path was set up, the measurement was started with the 655-nm laser to directly excite the Er^3+^ ions, and the emission was monitored at 1532 nm. The laser power was increased from low to high. At each power, ten measurements were taken to determine the statistical error. After measuring with the 655-nm laser, the experiment was repeated using the 405-nm laser, which can only excite the Ir-tBuPBI complex. After the data were collected, the sample was replaced with a calibrated silicon detector to measure the excitation power density on the sample.

### J-V measurement

The device was placed into a homemade sample holder, and a series of steady voltage steps were applied on the device. At the same time, the current through the device was recorded. A B2902A Precision Source/Measure Unit was used to provide the voltage and measure the current.
